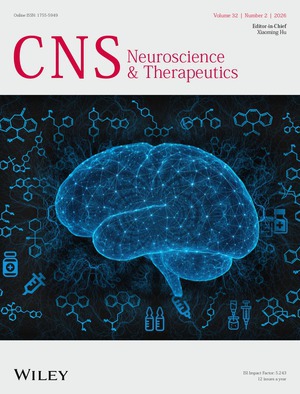# Front Cover

**DOI:** 10.1002/cns.70779

**Published:** 2026-02-04

## Abstract

Cover image: The cover image is based on the article *Effect of Anticholinergic Drug Burden on Postoperative Delirium in Elderly Patients: A Nested Case–Control Study* by Ting Zhang et al., https://doi.org/10.1002/cns.70731.